# Before and after the bottleneck: pediatric group A streptococcal infections in Houston, TX, from 2013 to 2023

**DOI:** 10.1128/spectrum.00308-26

**Published:** 2026-03-25

**Authors:** Misu A. Sanson, Maria Gabriela Segura, Aya Aboulhosn, Luis A. Vega, Sakina Chinwala, Lauren M. Sommer, J. Chase McNeil, Anthony R. Flores

**Affiliations:** 1 Division of Pediatric Infectious Diseases, Department of Pediatrics, Monroe Carell Jr. Children’s Hospital at Vanderbilt, Vanderbilt University Medical Center 12328, Nashville, Tennessee, USA; 2 Division of Infectious Diseases, Department of Pediatrics, McGovern Medical School at UTHealth Houston and Children’s Memorial Hermann Hospital 12339 , Houston, Texas, USA; 3 Division of Pediatric Infectious Diseases, Department of Pediatrics, Baylor College of Medicine, Texas Children’s Hospital 3989, Houston, Texas, USA; The Ohio State University College of Dentistry, Columbus, Ohio, USA

**Keywords:** group A *Streptococcus*, *Streptococcus pyogenes*, COVID-19, *emm *type

## Abstract

Toward the end of the COVID-19 pandemic, many reports described increasing group A streptococcal (GAS) infections. We sought to describe pandemic-associated changes in GAS clinical and molecular epidemiology. GAS isolates were collected from Texas Children’s Hospital laboratory from 01 January 2013 to 31 December 2023 and grouped into pre-pandemic (2013–2019) and pandemic (2020–2023) periods. Invasive (INV), skin and soft tissue infection (SSTI), and pharyngeal (PHG) disease types were determined by medical record review. All isolates were *emm* typed, and nonsusceptibility (NS) to tetracycline, erythromycin, and clindamycin was determined using standard methods. We analyzed 2,717 isolates (pre-pandemic, *n* = 1,640; pandemic, *n* = 1,077). Year-to-year variation was observed, but the highest GAS disease burden occurred in 2022 and 2023. Comparing disease types, we observed a subtle but significant increase in the frequency of GAS strains derived from INV infections. Relative to pre-pandemic, *emm*12 and *emm3* GAS showed significant increases in frequency, while *emm89*, *emm4*, and *emm6* showed concomitant decreases. Second-line antimicrobial NS increased during the pandemic period but was transient with a rapid return to pre-pandemic rates in 2023. The pandemic-related surge in GAS disease in the Houston, TX, pediatric population was associated with changes in the frequency of GAS *emm* types and a small shift toward invasive disease. NS to second-line antimicrobials fluctuated during the pandemic and differed substantially from GAS derived from adult infections. Continued surveillance will be paramount to monitor ongoing changes in GAS epidemiology in pediatric populations.

**IMPORTANCE:**

Group A *Streptococcus* (GAS) causes common childhood infections such as strep throat but can also lead to severe, life-threatening disease. During and after the COVID-19 pandemic, many regions reported sharp increases in GAS infections, yet the reasons for these changes were unclear. Using long-term surveillance of children in a large US city, this study shows that the post-pandemic rise in GAS was accompanied by meaningful changes in both the types of infections and the strains causing them. We found a small but significant increase in invasive disease, shifts in the most common GAS strain types, and temporary increases in resistance to certain antibiotics. These changes likely reflect how pandemic-related disruptions altered bacterial spread. By examining both mild and severe infections over more than a decade, this work demonstrates how large-scale changes in human behavior can reshape bacterial disease patterns and highlights the importance of continued monitoring to protect child health.

## INTRODUCTION

*Streptococcus pyogenes* (group A *Streptococcus*, GAS) is a gram-positive, human-adapted bacterial pathogen and a major cause of pediatric infections worldwide. GAS infections may result in a wide spectrum of disease, including the relatively benign pharyngitis (“strep throat”) and superficial skin infections (e.g., impetigo). GAS may also result in more severe invasive (iGAS) infections such as cellulitis, necrotizing fasciitis (“flesh-eating disease”), and streptococcal toxic shock syndrome associated with high morbidity and mortality (1). Following the dramatic worldwide increase in iGAS observed in the late 1980s, many countries initiated national-level surveillance to monitor the epidemiology of iGAS (2, 3). In addition, smaller, local surveillance reports have augmented country-level surveillance in important ways, such as the inclusion of non-invasive GAS disease (e.g., pharyngeal [PHG] and superficial skin infections) (4, 5). Both local and large-scale surveillance have been central in the identification of critical changes in iGAS disease epidemiology, including the emergence of novel GAS clones (6–9).

The COVID-19 pandemic was associated with dramatic shifts in the incidence of many respiratory pathogens, including GAS. Early in the pandemic, a decreased frequency of bacterial pathogens such as GAS and *Streptococcus pneumoniae* was observed (10, 11). Presumably, this was due to, at least in part, the implementation of nonpharmaceutical interventions (NPIs) such as school closures, social distancing, and mask mandates (12). Subsequent relaxation of pandemic-specific NPIs saw the dramatic rise of many pediatric infections of both viral (e.g., respiratory syncytial virus [RSV] [13]) and bacterial (e.g., GAS [14–17]) etiologies.

Our group has conducted GAS disease surveillance in the Houston metropolitan area for over 13 years. Using a combination of clinical and GAS strain molecular epidemiology, our studies have augmented national-level surveillance (Active Bacterial Core surveillance, ABCs) of iGAS by the Centers for Disease Control and Prevention (CDC) through the inclusion of PHG and superficial skin and soft tissue infections (SSTIs) in addition to iGAS (4, 18). Consistent with ABCs reports (19), prior to the COVID-19 pandemic, we showed an overall increase in pediatric GAS disease that was attributable to increased frequency of GAS strains with nonsusceptibility (NS) to second-line antimicrobials (e.g., erythromycin and clindamycin) (20). However, beginning in 2020, we reported significantly reduced pediatric iGAS-associated hospitalizations (10) only to be followed by a rapid resurgence of GAS disease in the last quarter of 2022 (15) that mirrored outbreaks in multiple jurisdictions (14, 16, 17, 21). In this report, we seek to better define the changes in pediatric GAS disease clinical and molecular epidemiology associated with the COVID-19 pandemic in Houston, TX.

## MATERIALS AND METHODS

### Surveillance

Pediatric (ages 0–18 years) GAS isolates included in the study were collected between 1 January 2013 and 31 December 2023, under a protocol approved by the institutional review board at Baylor College of Medicine. Strains collected between 1 July 2013 and 30 June 2017 were previously reported (4) and were included in the current study and analyses. All GAS isolates from Texas Children’s Hospital (TCH) in the Texas Medical Center (TMC) and two satellite campuses were identified and processed at the main hospital in the TMC, which facilitates the collection of GAS isolates from all encounters. All strains were grown and stocked using standard procedures (22). Individual medical encounters were reviewed to determine the diagnosis and to categorize GAS (PHG, SSTI, or invasive [INV]) as we have previously described (4). GAS isolates obtained from disease prior to 1 January 2020 were defined as “pre-pandemic,” whereas those obtained on or after 1 January 2020 through 31 December 2023 were defined as “pandemic.”

### Group A *Streptococcus* isolates *emm* typing and antimicrobial susceptibility testing

Genomic DNA was extracted from individual GAS strains, and *emm* typing was performed using the standard protocol from the CDC *Streptococcus* reference laboratory (23). All available GAS strains were subjected to antimicrobial susceptibility testing using disk diffusion per Clinical Laboratory Standards Institute (CLSI) guidelines (24). Antimicrobials assayed included clindamycin (CC), tetracycline (TE), and erythromycin (E). Interpretations (susceptible [S], intermediate [I], or resistant [R]) were made using CLSI breakpoints for β-hemolytic streptococci (24). NS was defined as strains exhibiting an intermediate or resistant phenotype to the tested antimicrobial. All strains defined as intermediate through disk diffusion were further analyzed using MIC test strips (Liofilchem, Fisher Scientific), and susceptibility was reinterpreted using CLSI breakpoints for β-hemolytic streptococci (24).

### Statistics

Statistical calculations were performed using Prism (GraphPad, San Diego, CA, USA). Chi-square or Fisher’s exact test was used to compare categorical variables. Student’s *t*-test was used to compare continuous variables. A *P* value of ≤0.05 (following Bonferroni correction for multiple comparisons) was considered significant for all comparisons.

## RESULTS

**Fig 1:**
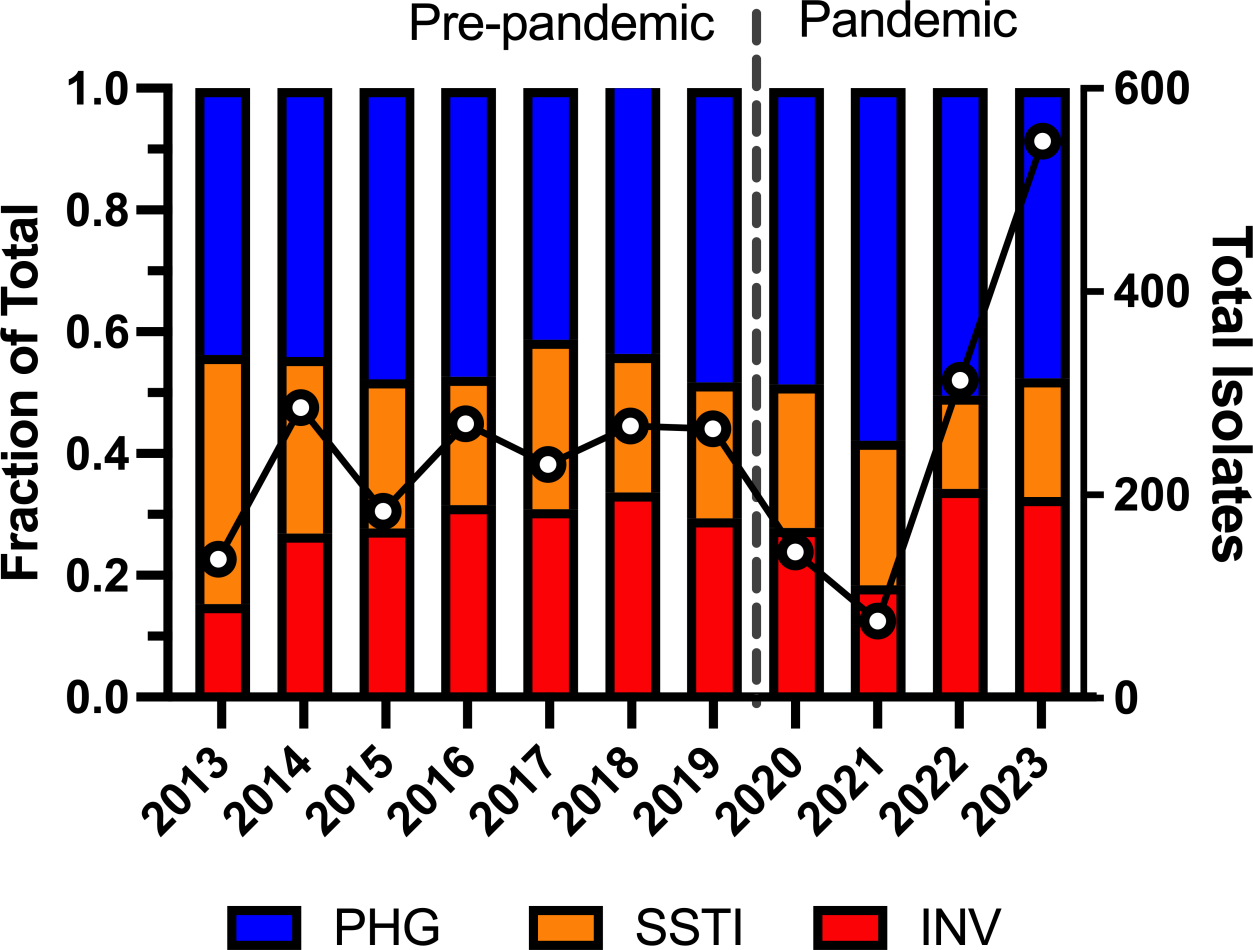
Annual total (right *y*-axis) and distribution of GAS isolates by disease type [PHG, SSTI, or INV] (left *y*-axis). Pre-pandemic (2013–2019) and pandemic (2020–2023) periods are labeled and separated by a vertical dashed line.

A total of 2,717 non-duplicate GAS isolates (*n* = 1,640 pre-pandemic and *n* = 1,077 pandemic) were available for analysis. Prior to the pandemic, the mean annual number of GAS cases from all causes was 234 (95% CI, 183–285). Year-to-year variation was observed, with the lowest total in 2021 (*n* = 76) and highest in 2023 (*n* = 548)—nearly double the mean number of GAS cases prior to 2020 (Fig. 1). Complete clinical and demographic information was available for 2,508 GAS cases (*n* = 1,530 pre-pandemic and *n* = 978 pandemic) (Table 1). Overall, GAS derived from pharyngeal infection accounted for nearly half of all collected isolates in both periods (Fig. 1; Table 1). No significant differences in age or sex were observed between the two periods (Table 1). Comparing the pre-pandemic relative to the pandemic distribution of GAS strain disease type, we observed subtle changes in the frequency of GAS derived from SSTI (24.2% versus 20.6%, *P* = 0.032) and GAS derived from invasive disease (30.1% versus 34.2%, *P* = 0.039) (Fig. 1; Table 1). Among GAS isolates categorized as SSTI, the frequency of individual diagnoses differed between the two periods (*P <* 0.001, chi-square test) and was attributable to a rise in the frequency of diagnoses defined as skin abscess (34.8% versus 49.8%, *P* = 0.002) with a concomitant decrease in cellulitis (39.1% versus 27.4%, *P =* 0.02) (Table 1). Similarly, the distribution of invasive diagnoses differed between the pre-pandemic and pandemic periods (*P* < 0.001, chi-square test) but could not be attributed to a single diagnosis. Previous reports of pediatric GAS infections following the pandemic showed increased cases of pneumonia (25, 26). We also observed increased frequency of pneumonia diagnoses in the pandemic period (8.7% versus 11.4%), but this was not significant (Table 1). Interestingly, we observed a more than twofold decrease in the frequency of osteomyelitis/septic arthritis diagnosis in the pandemic period compared to the pre-pandemic period, but did not meet the threshold for significance following correction for multiple comparisons (*P* = 0.054) (Table 1).

**TABLE 1:** Characteristics of GAS cases at Texas Children’s Hospital: pre-pandemic and pandemic periods^*g*^

	Pre-pandemic (2013–2019)			Pandemic (2020–2023)			
Disease/infection type^*a*^	Cases (*n* [%])	Male sex (*n* [%])	Patient age (mean [range]) (years)	Cases (*n* [%])	Male sex (*n* [%])	Patient age (mean [range]) (years)	*P* value
PHG	698 (45.6)	377 (54.0)	7.6 (0.3–17.7)	443 (45.3)	245 (55.3)	7.4 (0.8–17.8)	NS
SSTI	371 (24.2)	221 (59.6)	6.6 (0.02–17.7)	201 (20.6)	106 (52.7)	7.3 (0.1–18.0)	0.032^*b*^
Cellulitis	145 (39.1)	86 (59.3)	6.6 (0.03–17.7)	55 (27.4)	29 (52.7)	6.8 (0.2–18.0)	<0.001^*c*^
Abscess (skin)	129 (34.8)	85 (65.9)	7.5 (0.3–17.6)	100 (49.8)	47 (47.0)	8.0 (0.1–17.9)	
Rash	97 (26.1)	51 (52.6)	5.7 (0.02–17.2)	46 (22.9)	24 (52.2)	6.2 (0.1–15.4)	
INV	461 (30.1)	258 (56.0)	6.7 (0.08–18.0)	334 (34.2)	188 (56.3)	7.1 (0.01–18.0)	0.039^*b*^
Abscess							
Peritonsillar	81 (17.6)	39 (48.1)	10.6 (3.1–18.0)	68 (20.4)	24 (35.3)	10.4 (0.4–18.0)	<0.001^*c*^
Retropharyngeal	54 (11.7)	28 (51.9)	4.9 (0.1–14.1)	35 (10.5)	19 (54.3)	5.4 (0.7–15.6)	
Lymph node	34 (7.4)	24 (70.6)	4.7 (0.9–17.3)	21 (6.3)	15 (71.4)	3.3 (0.2–10.5)	
OM/mastoiditis	90 (19.5)	47 (52.2)	5.4 (0.08–18.0)	60 (18.0)	40 (66.7)	6.3 (0.09–13.9)	
Osteomyelitis/SA	51 (11.1)	30 (58.8)	6.8 (0.05–17.8)	18 (5.4)	11 (61.1)	6.8 (0.04–16.3)	0.054^*d*^
Septic shock/STSS	28 (6.1)	16 (57.1)	6.3 (0.1–16.9)	16 (4.8)	8 (50.0)	6.33 (0.5–17.6)	
Pneumonia/empyema	40 (8.7)	22 (55.0)	6.1 (0.04–16.8)	38 (11.4)	24 (63.2)	6.1 (0.7–16.2)	
Bacteremia^*e*^	19 (4.1)	15 (78.9)	5.9 (0.8–14.0)	19 (5.7)	13 (68.4)	6.2 (0.01–14.6)	
Necrotizing fasciitis	2 (0.4)	1 (50.0)	9.9 (3.2–16.6)	3 (0.9)	3 (100)	11.4 (3.1–15.6)	
SSTI (invasive)^*f*^	18 (3.9)	10 (55.6)	5.8 (0.09–15.1)	25 (7.5)	13 (52.0)	6.4 (0.9–6.7)	
Other	44 (9.5)	27 (61.4)	7.3 (0.5–17.8)	31 (9.3)	21 (67.7)	7.4 (0.1–16.8)	
Total	1,530	856 (55.9)	7.1 (0.02–18.0)	978	562 (55.1)	7.2 (0.05–18.0)	

^*a*^ Disease/infection type determined by discharge diagnosis and chart review as described in Materials and Methods.

^*b*^ *P* values comparing frequency of disease type between the two periods determined by Fisher’s exact test.

^*c*^ *P* values comparing distribution of infection types within SSTI or INV disease determined by chi-square test.

^*d*^ *P* value comparing frequency of osteomyelitis/SA between the two periods determined by Fisher’s exact test with Bonferroni correction for multiple comparisons.

^*e*^ Bacteremia without a focus.

^*f*^ SSTI disease (cellulitis, skin abscess, or rash) otherwise defined as invasive (e.g., concomitant bacteremia).

^*g*^ GAS, group A *Streptococcus*; OM, otitis media; SA, septic arthritis; STSS, streptococcal toxic shock syndrome; NS, not significant.

**Fig 2:**
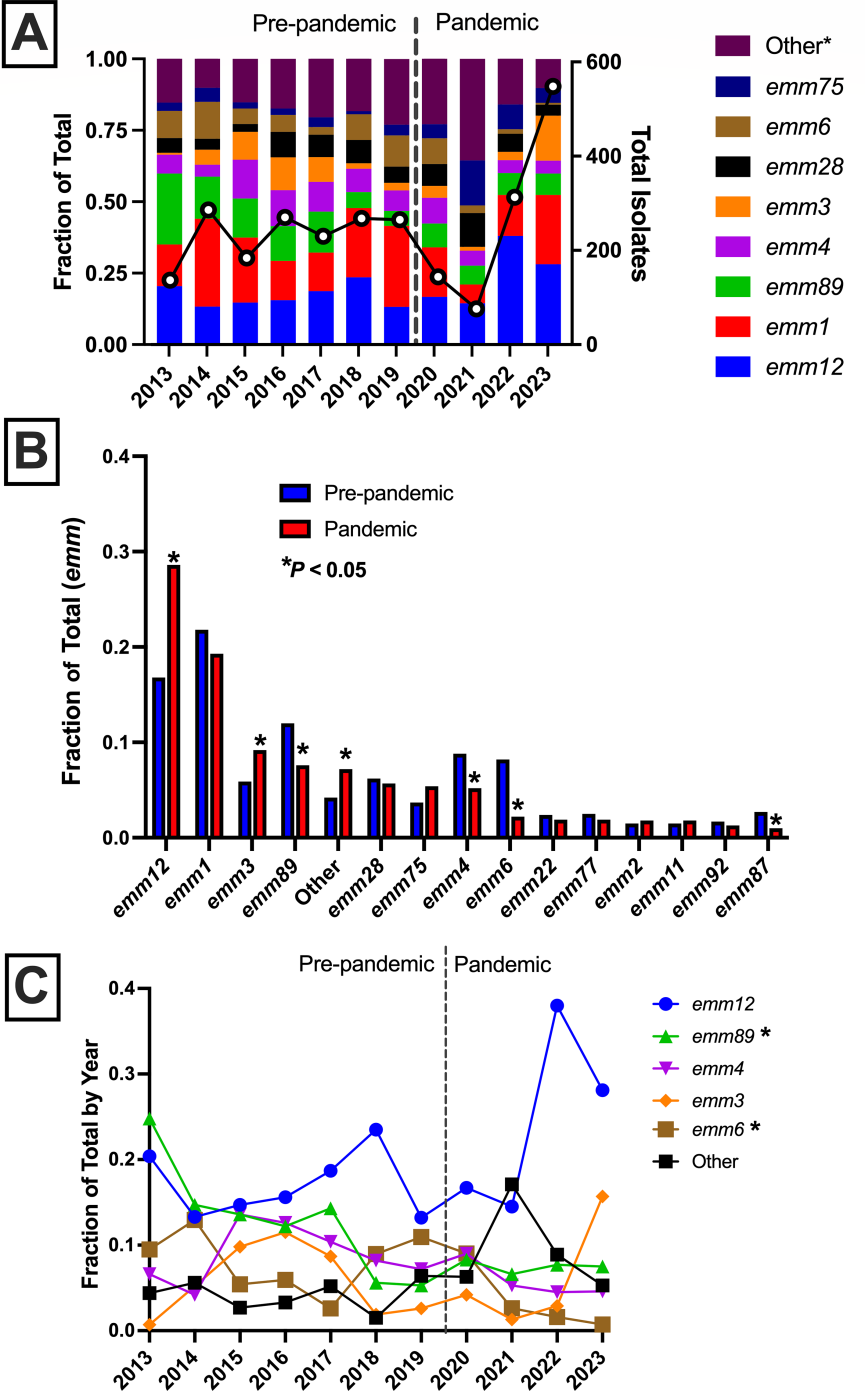
Changes in pediatric GAS strain *emm* types by year, 2013–2023. (**A**) Frequency of most common GAS *emm* types (left *y*-axis, right-hand key) by year and annual total (right *y*-axis, white dots with solid line) as in Fig. 1. Other* indicates *emm* types not otherwise indicated in key, regardless of the total number in either period. (**B**) Comparison of GAS *emm* type frequency in pre-pandemic (blue bars) and pandemic (red bars) periods. Asterisks (*) indicate a *P* value <0.05 (Fisher’s exact, Bonferroni corrected). Other represents the total “rare” (<10 observations in either pre-pandemic or pandemic periods) *emm* types. (**C**) Changes in selected GAS *emm* type frequency by year. Asterisk (*) indicates a significant (*P* < 0.05, linear regression) change over the entire 11-year period (2013–2023). Pre-pandemic (2013–2019) and pandemic (2020–2023) periods are labeled and separated by a vertical dashed line.

Given the previously reported increased frequency of *emm12* GAS associated with an upsurge of cases in late 2022 (15), we next sought to compare the distribution of GAS *emm* types between the two periods (pre-pandemic versus pandemic). We observed substantial year-to-year changes in frequency for the most common GAS *emm* types in our analysis (Fig. 2A). However, comparing the *emm* type frequency between the pre-pandemic and pandemic periods revealed important changes in the frequency of several GAS *emm* types (Fig. 2B). Increased frequency in the pandemic period relative to the pre-pandemic was seen for GAS of type *emm12* (16.8% versus 26.8%, *P <* 0.001), consistent with our previous report (15), and in *emm3* (5.9% versus 9.4%, *P* = 0.012). Concomitant decreased frequency was seen in *emm89* (12.0% versus 7.3%, *P <* 0.001), *emm4* (8.8% versus 4.8%, *P <* 0.001), and *emm6* (8.2% versus 1.9%, *P <* 0.001) (Fig. 2B). Overall diversity of *emm* types increased as well, as indicated by the increase in “Other” *emm* types (collectively rarer *emm* types defined as <10 observations) during the pandemic period (Fig. 2B). Interestingly, GAS defined as an “other” *emm* type was distributed differently among disease types in the pandemic compared to the pre-pandemic period (*P* = 0.0003, chi-square test), with iGAS being more common in the pandemic period (17.4% versus 33.3%). To further assess changes in GAS *emm* type frequency over time, we plotted the annual frequency of *emm12*, *emm89*, *emm4*, *emm3*, and *emm6* GAS for comparison (Fig. 2C). In the pre-pandemic period, *emm89* GAS showed a substantial decline (*R*^2^ = 0.8, *P* = 0.007, linear regression) from a high of 24.8% in 2013 to a nadir of 5.3% in 2019 followed by stabilization during the pandemic period. Similarly, *emm6* GAS showed a sustained decline over the 11-year time frame from a peak of 12.9% in 2014 to a low of 0.7% in 2023 (*R*^2^ = 0.366, *P* = 0.049). Also of note, while *emm12* GAS showed a significant increase in frequency during the pandemic period, the overall trend over the 11-year period was not significant and included a substantial drop in 2023 (28.1%) from a high of 38.0% in 2022 (Fig. 2C).

**Fig 3:**
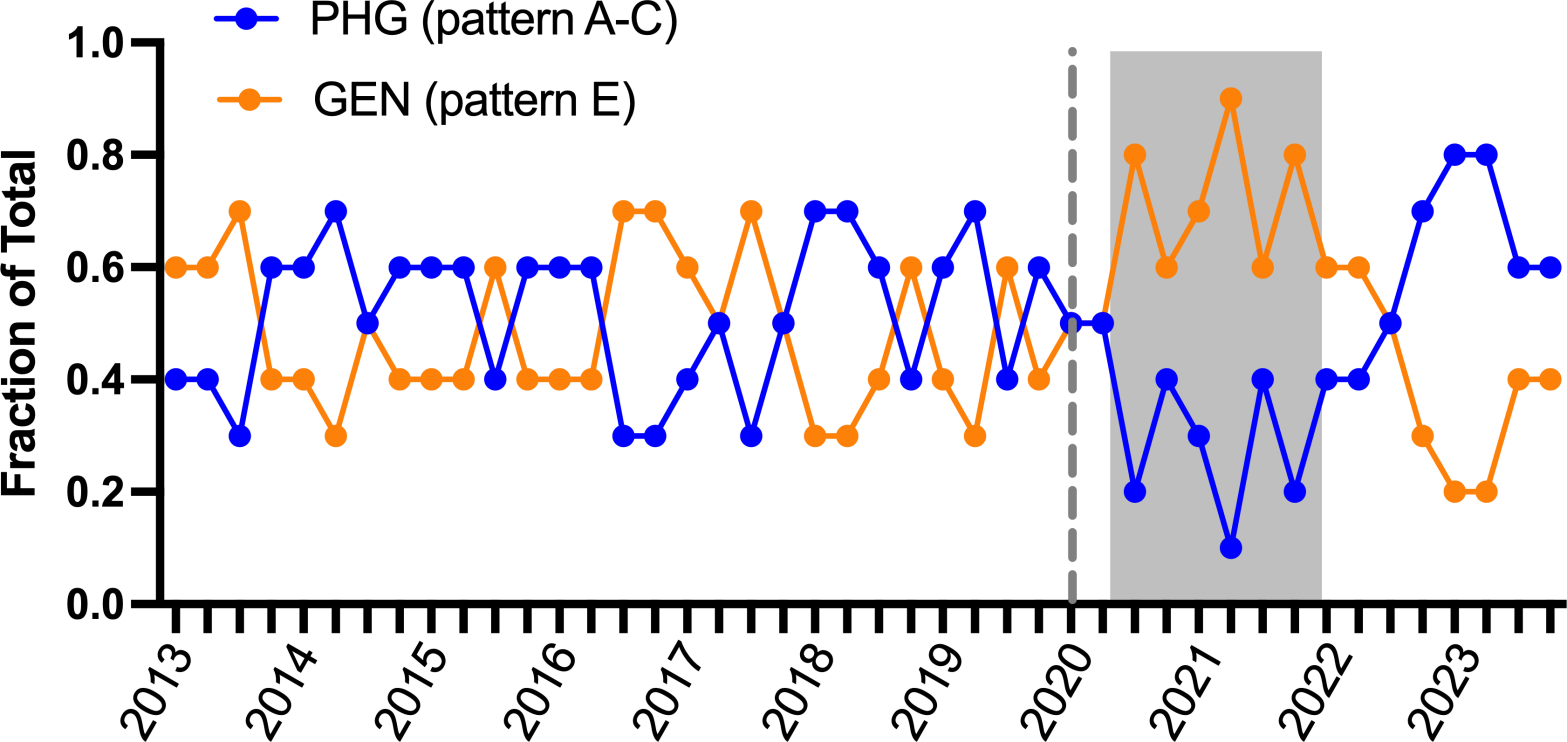
Shifts in GAS *emm* pattern by quarter, 2013–2023. GAS *emm* types were grouped as either pattern A–C (throat “specialists,” PHG) or pattern E (“generalists”) as described in the text. Proportions of pattern A–C and E GAS were plotted in 3-month intervals (quarters) over the entire study period. Pre-pandemic and pandemic periods are separated by a vertical dashed line. Shaded box indicates the period of intense NPI implementation (social distancing, masking, and school closures) for most locales during the pandemic (approximately April 2020 to December 2022).

GAS *emm* types may be grouped (i.e., *emm* patterns) based on the distinct chromosomal gene arrangement of *emm* and *emm-*like genes (27, 28). Three unique patterns (A–C, D, and E) are generally recognized with defined niche preferences: pattern A–C GAS (e.g., *emm1*, *emm6*, and *emm12*) is more frequently isolated from pharyngitis, pattern D from skin infections, and pattern E (e.g., *emm4*, *emm28*, and *emm89*) found equally among both throat and skin infections (so-called “generalists”) (29). Pattern D strains are rare in the United States and accounted for <1% of GAS strains (e.g., *emm43*, *n* = 10) in our surveillance. Inasmuch as infection site tropism (throat versus skin) may influence the mode of transmission (droplet versus direct contact, respectively), we hypothesized that implementation of NPIs during the pandemic directly influenced the distribution of *emm* patterns. More specifically, we hypothesized that NPIs would have a greater negative effect on the transmission of pattern A–C compared to pattern E GAS strains. After grouping *emm* types into pattern A–C (throat “specialists”) and E (generalists) (30), we examined the changes in frequency in 3-month intervals (quarters) over the study period (2013–2023) (Fig. 3). Prior to the pandemic, we observed variation consistent, at least in part, with previously reported seasonal changes in *emm* pattern distribution (4, 31). However, beginning in Q3 of 2020, pattern E (generalists) *emm* types predominated until early 2022 (Fig. 3, shaded box). We also observed a “rebound” in pattern A–C GAS strains that coincided with the marked increase in GAS disease in 2022 and 2023 (Fig. 1 and 2A).

**Fig 4:**
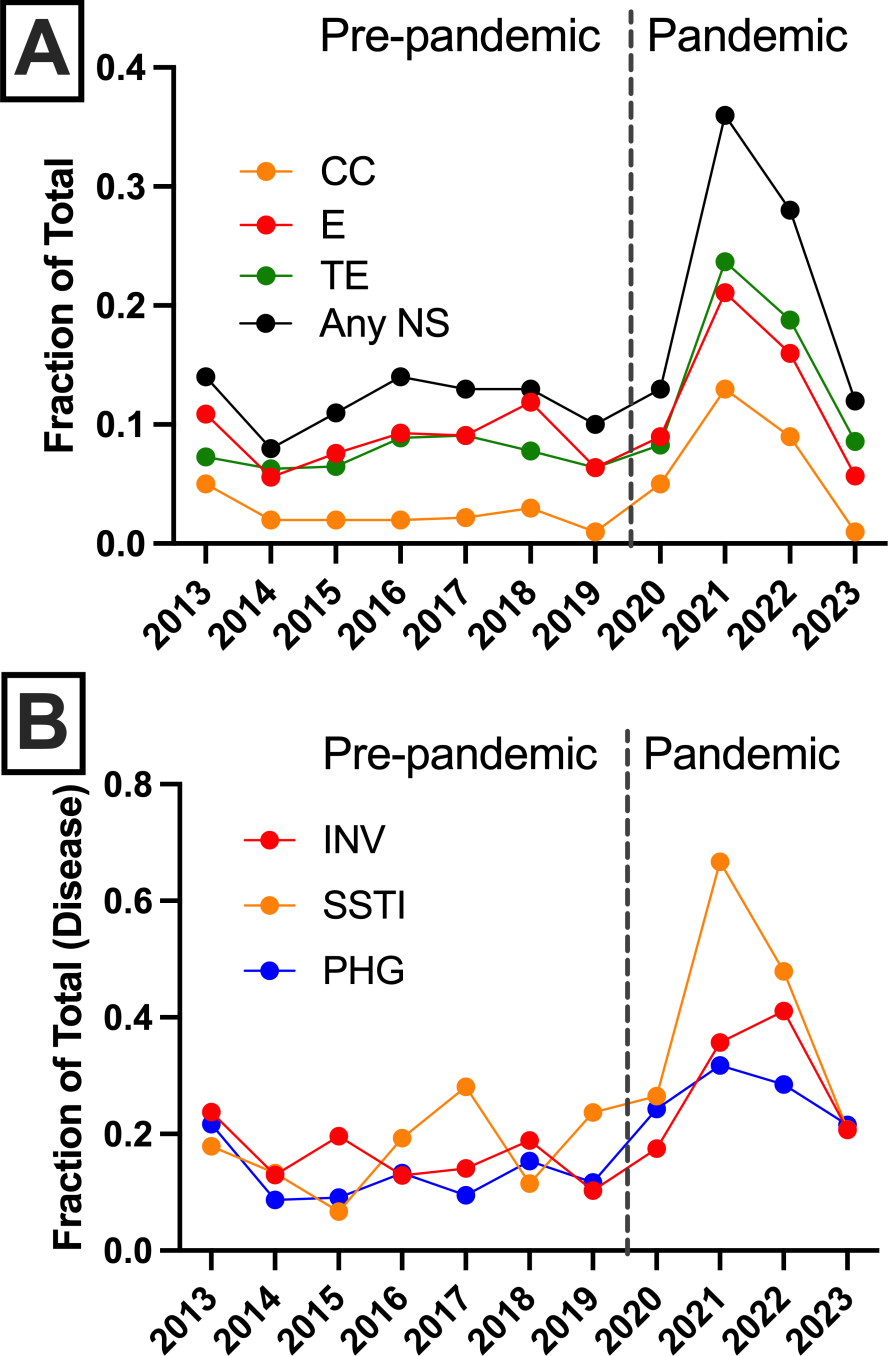
Changes in pediatric GAS strain NS to second-line antimicrobials by year, 2013–2023. (**A**) Frequency of NS to clindamycin (CC), erythromycin (E), tetracycline (TE), or any antimicrobial (Any) by year for all pediatric GAS strains. (**B**) Annual frequency of NS to any antimicrobial by disease type. Note that the large variation observed in 2021 coincides with the lowest (*n* = 76) number of GAS isolates. Pre-pandemic (2013–2019) and pandemic (2020–2023) periods are labeled and separated by a vertical dashed line.

Multiple GAS surveillance reports in the US have demonstrated increased frequency of NS to second-line antimicrobials over the past 10 years (18, 20, 32). When comparing the two study periods, we found that the overall NS to any antimicrobial (clindamycin, erythromycin, and/or tetracycline) increased from 11.6% to 18.3% (*P* < 0.0001). Examination of NS by year showed the increase in NS to be transient and returned to the pre-pandemic baseline by 2023 (Fig. 4A). NS to any antimicrobial peaked in 2021 (36%), a year with the lowest total number of GAS strains collected (*n* = 76). Individually, we observed increases in NS to clindamycin (2.1% to 4.9%, *P* < 0.0001) and tetracycline (7.5% to 12.6%, *P* < 0.0001) during the pandemic compared to the pre-pandemic period. No significant differences in NS rates between disease types (INV, SSTI, or PHG) were observed between the two periods for any antimicrobial (Fig. 4B). While NS to any antimicrobial increased to >60% in SSTI in 2021 (Fig. 4B), this was not significant given the small number of observations (*n* = 76). The significant increase in NS during the pandemic period could not be attributed to any single *emm* type. Rather, the pandemic increase was more likely attributable to the overall higher frequency of NS to any antimicrobial among pattern E strains (268/1,160, 23.1%) compared to pattern A–C (137/1,509, 9.1%) over the 11-year period.

## DISCUSSION

As one of the largest reports of pediatric GAS disease in the United States, our study examined the changes in the epidemiology of pediatric GAS disease in the Houston metropolitan area prior to and during the COVID-19 pandemic. In addition to iGAS, our study leveraged comprehensive surveillance for non-invasive pediatric GAS disease to provide greater detail of pandemic-associated changes in pediatric GAS disease. Early pandemic years (e.g., 2020) were notable for markedly reduced GAS disease, only to be followed by a rapid and sustained surge in late 2022 that persisted into 2023. The rapid resurgence of GAS was associated with significant changes in the frequency of disease type, with a small but significant shift toward greater iGAS from SSTIs. Within cases of iGAS in pre-pandemic and pandemic periods, more subtle changes of infection types were noted but could not be individually attributed to the observed increase in iGAS during the pandemic period. The precise mechanism(s) contributing to increased GAS disease observed in our study remains uncertain but likely a combination of relaxation of COVID-19-related NPIs, post-pandemic changes in respiratory viral co-pathogens (12), and potential emergence of new GAS strain variants with increased transmissibility (33, 34). Taken together, these factors suggest that, at the population level, these processes may also reflect a period of reduced GAS exposure during 2020–2021, resulting in increased susceptibility among children (“immunity debt”), followed by renewed transmission after the relaxation of NPIs. Importantly, the resurgence observed in Houston occurred without evidence of a single GAS lineage, supporting a model in which altered host susceptibility and transmission dynamics permit polyclonal expansion rather than clonal replacement.

The current study augments our previous report of increased GAS disease in the same population in 2022 (15). In that study, one of the key elements of the Houston pediatric GAS epidemiology was the disproportionately increased representation of *emm12* GAS strains compared to pre-pandemic rates (15). In our updated analysis that includes 2023, *emm12* GAS strains remain elevated compared to prior years but decreased from the peak in 2022. A predominance of *emm12* GAS associated with post-pandemic surges in GAS infections has also been noted in the United States (35) and other countries (26, 35–37). However, to date, it is unclear if the increased frequency of *emm12* GAS is associated with novel strain emergence. Viewed alongside the broader *emm*-type shifts described above, post-pandemic increases in GAS disease appear to be driven by region-specific shifts in strain composition rather than a uniform global expansion of a single clone, underscoring the stochastic nature of GAS population structure following transmission bottlenecks.

Observed changes in GAS strain epidemiology (e.g., shifts in the frequency of GAS *emm* types pre-pandemic versus post-pandemic) in our investigation and previously published studies are indicative of transmission bottlenecks likely precipitated through NPIs. Within this framework, while clonal expansion of the M1_UK_ lineage has been implicated in surges of invasive GAS disease in the UK and parts of Europe (33, 34), such events appear to represent regional amplifiers rather than a universal post-pandemic paradigm. Our findings support a broader model in which altered transmission and susceptibility landscapes permit diverse GAS lineages to expand following relaxation of NPIs. In contrast to our observed *emm12* GAS emergence post-pandemic, other locales have identified changes in *emm1* GAS epidemiology as the main driver in surges of iGAS in late 2022 into 2023. Specifically, in the UK, the M1_UK_ clone became dominant with evidence of a unique M1_UK_ clonal expansion suggestive of a bottleneck associated with the COVID-19 pandemic (33). Transmission/genetic bottlenecks in association with the COVID-19 pandemic have also been observed with multiple seasonal respiratory viruses—e.g., influenza (38, 39) and RSV (40, 41) among others—many of which showed marked increases in frequency upon removal of pandemic NPIs (42, 43). Furthermore, population structures of a subset of RSV strains (RSV-B) formed geographically distinct clusters through genetic shift (44), akin to the regional differences observed in GAS molecular epidemiology post-pandemic. Thus, perhaps not surprisingly, the strain epidemiology in post-pandemic surges in GAS infections is reflective of the stochastic nature of renewed transmission upon the removal of pandemic NPIs. Similar patterns have been observed across multiple respiratory pathogens, in which suppression of circulation during the pandemic was followed by atypical seasonality, altered age distributions, and shifts in population structure once social mixing resumed.

**Fig 5:**
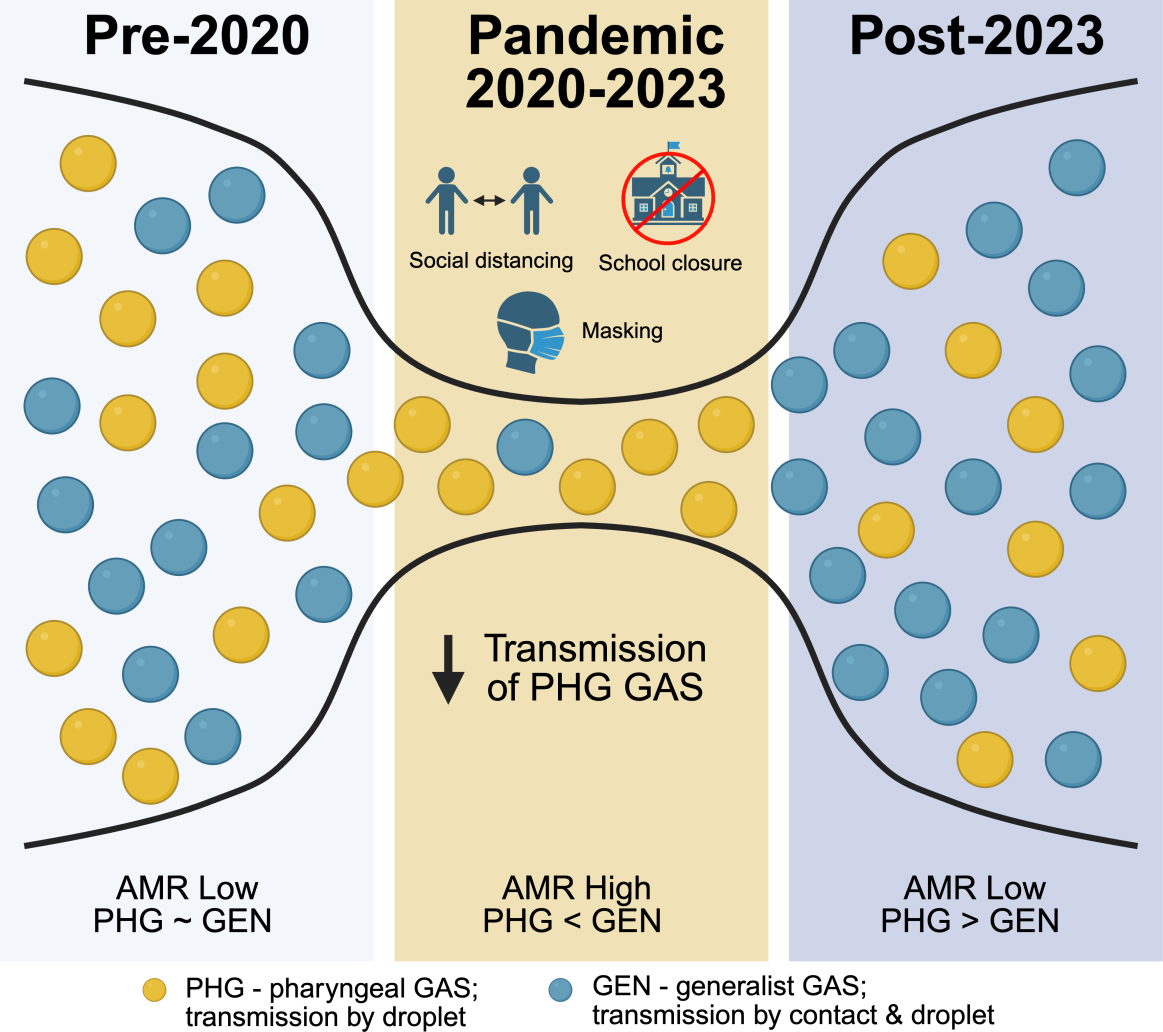
Model demonstrating plausible effect of a transmission bottleneck on GAS strain epidemiology before, during, and after the COVID-19 pandemic. Prior to the pandemic (pre-pandemic), pattern A–C (PHG, blue dots) and pattern E (GEN, yellow dots) GAS *emm* types were found in roughly equal proportions, and resistance to second-line antimicrobials was low. During the pandemic, NPIs (e.g., social distancing, school closures, and masking) disproportionately affected transmission of pattern A–C (PHG) GAS *emm* types. The effect was a transient increase in pattern E GAS strains with higher rates of second-line antimicrobial resistance. Following relaxation of NPIs (post-pandemic), transmission of pattern A–C GAS strains surged in a relatively non-immune pediatric population, resulting in a higher proportion of pattern A–C GAS strains that were less likely to harbor antimicrobial resistance (created in https://BioRender.com).

Our analysis showed a sustained shift in GAS strain epidemiology toward pattern E (generalists) and away from pattern A–C (throat specialists) early in the pandemic. The most likely explanation for this shift is the implementation of NPIs that reduced the major route of transmission (droplet) for pattern A–C strains (modeled in Fig. 5). Presumably, the ability of pattern E GAS strains to sustain transmission during the pandemic and the use of NPIs can be attributed to their more diverse transmission routes, given their propensity to infect both skin and throat (29). Seasonality in the incidence of iGAS caused by pattern A–C and E strains has previously been noted in our local surveillance and that of the ABCs (4, 31). The proportion of iGAS caused by pattern A–C strains generally increases during the winter months in the US, coinciding with increased incidence of respiratory viral disease (e.g., influenza and RSV). Co-infection with respiratory viruses is a known risk factor for GAS invasive disease (45, 46). Thus, another plausible explanation for the decrease in pattern A–C strains early during the COVID-19 pandemic is the concomitant decrease of respiratory viruses such as RSV and influenza during the same period (47). Congruent with this argument, the rapid increase in pattern A–C GAS disease following relaxation of NPIs coincided with the resurgence of respiratory viruses in geographically diverse locations (45, 46, 48).

We observed a significant increase in GAS nonsusceptibility to second-line antimicrobials early in the pandemic, followed by a return to pre-pandemic levels. When interpreted in the context of longer-term surveillance, this transient pediatric increase contrasts with sustained rises in macrolide and clindamycin resistance among iGAS infections nationally, particularly in adult populations. These differences likely reflect distinct transmission networks and risk factors in pediatric versus adult populations rather than intrinsic differences in GAS biology. GAS resistance to macrolide antibiotics is listed as a “concerning threat” in the most recent threat report published by the CDC (32), and national-level surveillance through the ABCs has shown a progressive increase in resistance to both clindamycin and macrolides (erythromycin). Currently, one-third of all iGAS recovered through the CDC ABCs are resistant to both clindamycin and erythromycin (49). Overall, our surveillance shows a much lower rate of resistance to clindamycin and erythromycin (3.2% and 9.2%, respectively). Second-line antimicrobial resistance is driven by a small subset of GAS *emm* types with high rates of resistance (e.g., *emm11*, *emm75*, *emm77*, and *emm92*) (18, 20), many of which may be encountered more frequently in high-risk groups (e.g., homeless, injection drug use) (20, 49, 50). Thus, the difference in resistance rates may be explained by the comprehensive surveillance (i.e., both invasive and non-invasive disease) derived from a pediatric population in a single metropolitan area (Houston, TX). Continued surveillance will be essential to monitor trends in second-line antimicrobial resistance in pediatric populations.

Clindamycin remains an important adjunctive therapy for iGAS infections secondary to inhibition of protein synthesis suppressing exotoxin production that attenuates toxin-mediated inflammation independent of bacterial burden. Against the backdrop of rising resistance nationally, preservation of therapeutic strategies that target toxin production represents an increasingly constrained but critical component of iGAS management. Erosion of clindamycin activity has implications not only for individual patient management but also for outbreak control and empiric treatment strategies. Multiple studies demonstrate improved clinical outcomes with combination clindamycin and beta-lactam therapy (51–56) and form the basis of current guideline recommendations. As clindamycin resistance continues to rise, preserving strategies that target toxin production remains critical. Thus, alternative protein synthesis inhibitors such as oxazolidinones (linezolid and tedizolid) may need to be considered (54) as adjunctive therapy in iGAS disease.

Our study has notable limitations. Our surveillance includes a single hospital system (Texas Children’s Hospital) and metropolitan area (Houston, TX). However, TCH serves as a major referral center for all 51 associated outpatient clinics and includes a catchment area of ~2 million children, making our collection representative of the greater Houston pediatric population. It is possible that some GAS cases defined as pharyngeal represent carriage despite the chart review to include only those GAS isolates from patients with symptoms. We were also unable to assess viral co-infection due to the inconsistent testing performed on patients evaluated for GAS disease. Finally, we encountered several isolates classified as “intermediate” using disk diffusion, most commonly for clindamycin. For most isolates, the intermediate phenotype to clindamycin was resolved using antimicrobial test strips. Thus, it is possible that some isolates with inducible clindamycin resistance were misclassified, and our analysis underestimated clindamycin nonsusceptibility.

### Conclusion

The COVID-19 pandemic altered the epidemiology of multiple pathogens including GAS. Our study provides a comprehensive analysis of the GAS disease and strain epidemiology in the pediatric population in Houston, TX, prior to and during the pandemic. Importantly, through the inclusion of non-invasive GAS disease (i.e., pharyngeal and superficial skin infections), we were able to demonstrate small but significant shifts in GAS disease toward greater iGAS during the pandemic period. Increased GAS disease was associated with significant shifts in GAS strain *emm* types in addition to increased nonsusceptibility to second-line antimicrobials. Future work should include elucidation of GAS genomic epidemiology to better define changes in GAS population structure, including the emergence of antimicrobial resistance genetic elements. More broadly, the increasing incidence and severity of GAS disease observed in multiple high-income settings underscore the need for improved preventive strategies, including continued evaluation of GAS vaccine candidates. Important differences exist in GAS derived from pediatric and adult populations that warrant continued monitoring.
